# *Zanthoxylum* Species: A Review of Traditional Uses, Phytochemistry and Pharmacology in Relation to Cancer, Infectious Diseases and Sickle Cell Anemia

**DOI:** 10.3389/fphar.2021.713090

**Published:** 2021-09-15

**Authors:** Innocent Uzochukwu Okagu, Joseph Chinedum Ndefo, Emmanuel Chigozie Aham, Chibuike. C. Udenigwe

**Affiliations:** ^1^ Department of Biochemistry, University of Nigeria, Nsukka, Nigeria; ^2^ Department of Science Laboratory Technology, University of Nigeria, Nsukka, Nigeria; ^3^ Natural Science Unit, School of General Studies, University of Nigeria, Nsukka, Nigeria; ^4^ School of Nutrition Sciences, University of Ottawa, Ottawa, ON, Canada

**Keywords:** Zanthoxylum, Ethnobotany, health benefits, phytochemicals, functional foods, Nutraceutials

## Abstract

The health benefits and toxicity of plant products are largely dependent on their secondary metabolite contents. These compounds are biosynthesized by plants as protection mechanisms against environmental factors and infectious agents. This review discusses the traditional uses, phytochemical constituents and health benefits of plant species in genus *Zanthoxylum* with a focus on cancer, microbial and parasitic infections, and sickle cell disease as reported in articles published from 1970 to 2021 in peer-reviewed journals and indexed in major scientific databases. Generally, *Z.* species are widely distributed in Asia, America and Africa, where they are used as food and for disease treatment. Several compounds belonging to alkaloids, flavonoids, terpenoids, and lignans, among others have been isolated from *Z.* species. This review discusses the biological activities reported for the plant species and their phytochemicals, including anticancer, antibacterial, antifungal, antiviral, anti-trypanosomal, antimalarial and anti-sickling properties. The safety profiles and suggestions for conservation of the *Z.* species were also discussed. Taken together, this review demonstrates that *Z.* species are rich in a wide range of bioactive phytochemicals with multiple health benefits, but more research is needed towards their practical application in the development of functional foods, nutraceuticals and lead compounds for new drugs.

## Introduction

Humans have continually depended on plants for food and medicine. Plants produce secondary metabolites in response to infective agents and environmental factors. Consequently, efforts have been made to isolate, characterize and investigate the beneficial effects of plant-derived secondary metabolites on human health. Notably, several bioactive compounds from plants have provided inspirations for the synthesis of chemical drugs, such as artesunates from artemisinins and quinolone antimalarials from quinine (Karunamoorthi et al., 2013; Lifongo et al., 2014; Pawar 2014; Numonov et al., 2019). Natural product chemists often rely on traditional knowledge on plants with medicinal potentials to produce crude extracts with biological activities. This process is followed by downstream processing to isolate the bioactive compounds, and structural characterization to identify them. In some cases, chemical modifications of the phytochemicals are used to produce more clinically effective and safer entities.

*Zanthoxylum* species*,* also known as *Fagara* species, have a long history of use as sources of food and drug by locals in different parts of Asia, America and Africa. In traditional medicine, many of the plant species are used in treating sickle cell anemia, trypanosomiasis, malaria and microbial infections, including tuberculosis and enteritis, with *Z. zanthoxyloides* Lam being the most reported species for these applications (Erichsen-Brown 1979; Burkill 1985). For example, fruits of *Z. lepreurii* Guill. and Perr. and Z. zanthoxyloides Lam are used in managing fever, malaria, tumors and sickle cell anemia (Tamdem 2019) while the stem bark, leaves, and roots are applied to suppress pain, and to treat arthritis, leprosy, stomachache and venereal diseases in Cameroon (Burkill 1998; Ngoumfo et al., 2010). Furthermore, different parts of *Z. lepreurii* are used to treat or manage tuberculosis, malaria, human immunodeficiency virus (HIV) and several types of bacterial infection in Uganda and other parts of Africa (Lamorde et al., 2010; Tabuti et al., 2010; Bunalema et al., 2014). In China and other parts of Eastern Asia, *Z. bungeanum* Maxim. (Syn. *Z. piperitum* Benn.) is widely used as a food condiment because of its perceived health benefits Hwang et al. (2008) and as cosmetics for maintaining skin quality (Hwang et al., 2020). In Chinese medicine, *Z. bungeanum* is used as spices and for treating infection and bone diseases (Lee and Lim 2008; Kim et al., 2017). The leaves, fruits and barks are used in treating bacterial and fungal infections, as spices, and for food preservation in Japan (Hatano et al., 2004). Similarly, different parts of *Z. schinifolium Siebold and Zucc.* are used as food condiments and for treating stomach pain, diarrhea, jaundice, and cold in Eastern Asia (Cui et al., 2009). Furthermore, herbal preparation from different parts of *Z. americanum* Mill. is traditionally used for treating tumors, fungal skin infections, respiratory, urinary, genital and gastrointestinal (GIT) diseases by herbal healers in Canada and United States (Moerman 1998). In Kanayatn Dayak Community, West Kalimantan, Indonesia, the stem and root of Z. bungeanum are consumed raw or after boiling in water to prevent alcohol intoxication and treat respiratory diseases (Sepsamli and Prihastanti 2019)*.* Other traditional and ethnobotanical uses of *Z.* species have been discussed elsewhere (Patiño et al., 2012; Adewole 2020; Lu et al., 2020a; Obakiro et al., 2020; Okagu et al., 2021). The objectives of this review are to discuss (1) the potential of *Z.* species as sources of bioactive phytochemicals that can be applied in the management and treatment of cancer, microbial and parasitic infections, and sickle cell disease; (2) chemical constituents involved in these biological activities; and (3) safety issues and suggestions for conservation of the plant species.

## Literature Search Strategy and Criteria for Selection of Articles

This study used a strategy similar to that reported by Nigussie et al. (2021). From repositories and search engines (PubMed, ScienceDirect, and Google Scholar), information related to the health benefits of *Z.* species, with emphasis on anticancer, anti-trypanosomal, antimicrobial, antiviral, antimalarial and anti-sickling properties, in peer-reviewed journals and ethnobotanical surveys published from 1970-July 3, 2021 were retrieved. The titles and abstracts of the studies were scanned using the inclusion criteria for this study. The search terms included cytotoxicity, anticancer, antimicrobial, antibacterial, anti-mycobacterial, antimalarial, antiviral, larvicidal, trypanocidal, anti-sickling and antiproliferative effect of *Zanthoxylum* species*, Fagara* species, and medicinal plants. In some cases, articles citing older papers and references of recent papers were used to obtain additional articles of interest. Studies reporting the biological activities of interest on different parts of *Z.* species including seeds, fruits, stem/stem bark, fruits, and root/root bark were included. Biological activities of crude extracts, their fractions and isolated compounds were also included. Where available, the mechanisms of action of the extract or isolated compounds were retrieved. Reviews, newspaper and other non-peer-reviewed articles were excluded. Similarly, studies reporting biological activities of *Z.* species other than those under consideration and in languages other than English were excluded. In this review, a test substance is considered bioactive when the outcome of the substance-treated group was substantial when determined qualitatively or quantitatively compared to controls (untreated group or group that received a standard drug).

The correctness of the scientific/botanical names of the plants reported in the included studies were confirmed with names available in botanical databases, including www.theplantlist.org, https://www.ipni.org/, https://www.ncbi.nlm.nih.gov/Taxonomy/Browser/, and https://www.tropicos.org. In cases where the plant name in the article was not the acceptable taxonomical nomenclature, the name in the botanical databases was used. A number of reviews have records of plant species in genus *Zanthoxylum*, including *Z. armatum* DC (Brijwal et al., 2013; Mukhtar and Kalsi 2018; Paul et al., 2018; Verma et al., 2021), *Z. limonella* (Supabphol and Tangjitjareonkun 2014), Z. nitidum (Roxb.) DC (Lu et al., 2020a), *Z. rhetsa* (Roxb.) DC (Maduka and Ikpa, 2021), and *Zanthoxylum bungeanum* Maxim (Zhang M. et al., 2017). Some of these reviews are not comprehensive, while others focused on health benefits related to metabolic diseases (Okagu et al., 2021) or the phytoconstituents such as alkaloids (Yuan et al., 2015; Wei et al., 2021), or were published in non-English languages (Zhang M. et al., 2017). In some previous reviews on traditional uses, only selected species were discussed with respect to a particular disease condition, e.g., Imaga (2010) on sickle cell anemia, Ochwang’i et al. (2014) on cancer, Sinan et al. (2019) on malaria, and Obakiro et al. (2020) on tuberculosis. These reviews were carefully analyzed and most of the reviewed studies were excluded from the present review. Hence, this review covers information on the phytochemistry and biological activities of interest for 25 plants species in Genus *Zanthoxylum*, namely Z. leprieurii Guill. and Perr., Z. bungeanum Maxim. (Syn. Z. nitidum Bunge; Z. piperitum Benn.; Z. bungeanum var. bungeanum; Z. simulans Hance); Z. schinifolium Siebold and Zucc., Z. clava-herculis L., Z. heitzii (Aubrév. and Pellegr.) P.G.Waterman, Z. chalybeum Engl., Z. ailanthoides Siebold and Zucc., Z. acanthopodium DC., Z. zanthoxyloides (Lam.) Zepern. and Timler, Z. paracanthum Kokwaro, Z. riedelianum Engl., Z. americanum Mill, Z. armatum DC. (Syn. Z. alatum Roxb.), Z. rhetsa DC., Z. buesgenii (Engl.) P.G.Waterman, Z. madagascariense Baker, Z. austrosinense C.C. Huang, Z. schreberi (J.F.Gmel.) Reynel ex C. Nelson (Syn. Z. monophyllum (Lam.) P. Wilson), Z. rhoifolium Lam., Z. davyi Waterm., Z. ovalifolium Tutcher, Z. fagara (L.) Sarg., Z. tingoassuiba A. St.-Hil., Z. gilletii (De Wild.) P.G.Waterman, and Z. poggei (Engl.) P.G.Waterman.

## *Zanthoxylum* Species as Potential Sources of Anticancer Agents

Cancer is a disease that is characterized by uncontrolled cell division and loss of contact inhibition, leading to formation of tumors. Cancers are resistant to apoptosis and develop angiogenic and metastatic potentials. Prevalence of cancer is rising worldwide, even in developing countries where the rise is partly due to adoption of the Western diet and sedentary lifestyle, and increase in the aging population, among other factors (Morounke et al., 2017). Cancer-related deaths are higher in economically poor countries due to late detection and poor access to treatment and support (India State-Level Disease Burden Initiative Cancer Collaborators, 2018). The number of cancer cases and cancer deaths globally are projected to increase geometrically in the future (Smittenaar et al., 2016), thus placing cancer as a major global health issue. A number of approaches, such as radiation therapies, chemotherapies and surgeries, or their combination, are available for cancer management and treatment. Radiation therapies and surgeries are effective but present cancer patients with discomfort. In most cases, chemotherapies are linked with side effects and some cancers are resistant to chemotherapy. Development of clinically potent cancer drugs that are selectively toxic to cancer cells without harming normal cells has become a public health priority. One promising strategy is to search for natural products with cancer cell-specific cytotoxicity. Some natural compounds from plants and marine organisms have promising applications as anticancer agents (Lichota and Gwozdzinski 2018). Traditionally, medicinal plants have been used for treating cancer in different parts of the world (Abubakar et al., 2020; Adewole 2020). Medicinal plants belonging to *Z.* species and their phytochemicals with anticancer properties are discussed below.

The potential of *Z.* species in the treatment of cancer has been assessed using both drug-sensitive and drug-resistant cancer cells. For example, antiproliferative activities have been demonstrated using cell culture studies for extracts of *Z. clava-herculis* L. stem bark against lung cancer (A549) (Wansi et al., 2009), *Z. ailanthoides* Seibold. and Zucc. stem against human colon cancer cell line (Colo 205) (Chou et al., 2011), *Z. heitzii* (Aubrév. and Pellegr.) P.G.Waterman fruits and barks against cervical cancer (HeLa), breast cancer (MCF-7), acute monocytic leukemia (THP-1) and human Caucasian prostate cancer (PC-3) (Dzoyem et al., 2013), *Z. leprieuii* and *Z. zanthoxyloides* fruits against PC-3, MCF-7, liver (WRL-68), and colon (Caco-2) (Misra et al., 2013), *Z. rhetsa* DC. stem bark and root bark against human stomach-cancer cell lines, SCL, SCL-6, SCL-3706, SCL-9, Kato-3, and NUGC-4 (Ahsan et al., 2014), *Z. chalybeum* Engl. and *Z. parachanthum* Kowaro stem bark against drug-sensitive and multidrug-resistant leukemia cell lines (CCRF-CEM and CEM/ADR5000) (Omosa et al., 2019), *Z. acanthopodium* DC*.* seed against MCF-7 cell line (Arsita et al., 2019), *Z. zanthoxyloides* roots against liver cancer (HCC), larynx cancer (HEp2) and breast cancer (BT549) (Andima et al., 2020), and *Z. paracanthum* root bark against human breast cancer (HCC 1395) and human prostate cancer (DU 145) cell lines (Kaigongi et al., 2020). Most of these reports are inspired by the traditional uses of the plant in managing health conditions, including cancer. Specifically, decoctions of different parts of *Zanthoxylum poggei* (Engl.) P. G. Waterman are ingested to treat tumors, among other health issues, in Cameroon and Congo (Wouatsa et al., 2013). Using a combination of chromatographic and spectrophotometric techniques, acridone and indoloquinazoline alkaloids, poggeicridone and 2-methoxy-7,8-dehydroruteacarpine, respectively were isolated from stem bark of the plant. On exposure to cultured PC-3 cells, the two alkaloids elicited significant cytotoxic effects with IC_50_ values of 15.8 and 22.1 μM, respectively compared to IC_50_ value of 0.9 μM for doxorubicin, the reference anticancer drug (Wansi et al., 2016).

**FIGURE 1:**
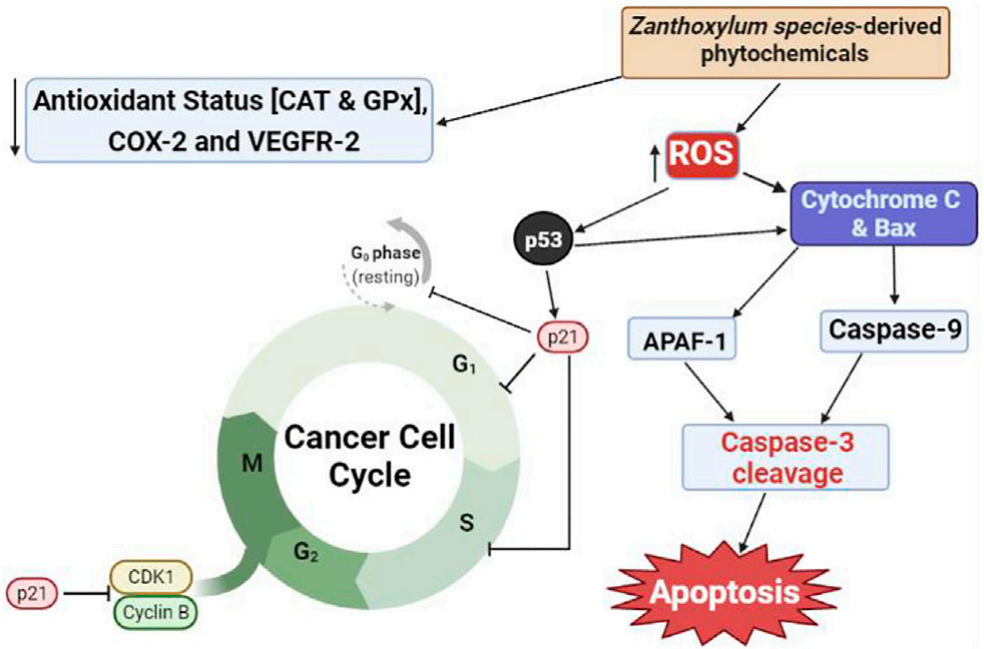
Proposed mechanism of anticancer activities of natural products from *Zanthoxylum* species. CAT, catalase; GPx, glutathione peroxidase; COX-2, cyclooxygenase-2; VEGFR-2, vascular endothelial growth factor receptor-2; CDK1, cyclin-dependent kinase-1; ROS, reactive oxygen species; Bax, Bcl-2-associated X protein; APAF-1, apoptosis protein activating factor-1.

These reports have provided support to the traditional uses of decoctions of *Z.* species alone or as a cocktail with other plant species for managing cancer. Most anticancer natural products act by blocking different mechanisms through which cancer cells grow, multiply and invade other cells, as well as resist the immune system. These mechanisms include the induction of cell cycle arrest, apoptosis and oxidative stress, as well as inhibition of angiogenesis, metastasis and growth signaling pathways in cancer cells (Ahmad et al., 2003; Michalkova et al., 2021). As shown in Figure 1, extracts of *Z.* species have been demonstrated to elicit anticancer properties via inhibition of angiogenesis Harahap et al. (2018) and DNA synthesis, induction of apoptosis Alam et al. (2017) and cell cycle arrest at Go/G1 Pieme et al. (2014) and G2-M phases, and suppression of cyclooxygenase (COX)-2 and vascular endothelial growth factor receptor type-2 (VEGFR-2) expression (Harahap et al., 2018); Table 1 presents the anticancer properties of crude extracts of *Z.* species whose anticancer constituents are unknown.

**TABLE 1:** Summary of *in vitro* anticancer properties of *Z.* species extracts.

Extract of *Z.* species	Range of concentration tested	Cancer model	Pharmacological effects	References
Ethanol extract of *Z. ailanthoides* stem	31.25–500 μg/ ml	Colo 205, Hep G2, B16-F1 and WEHI-3 cells	Suppressed cell viability by 46.4, 18.1, 9.2, and 5.2%, respectively. Extract induced apoptosis and cell cycle arrest at G2/M phase and increased ROS and Ca^2+^ levels, leading to cell damage.	Chou et al. (2011)
Ethyl acetate extract of *Z. acanthopodium* fruits	31.25–500 μg/ ml	4T1 breast cancer cells	Cytotoxic against cancer cells with IC_50_ value of 48.1 μg/ ml. Induced cell cycle arrest at G2/M phase and inhibited angiogenesis via suppression of gene expression of COX-2 and VEGFR-2.	Harahap et al. (2018)
Saponin-rich ethanol extract of *Z. armatum* DC fruit, bark and leaves	10–500 μg/ ml	Human breast cancer (MDA-MB-468 and MCF-7) and colorectal cancer (Caco-2) cells	At 200 μg/ ml, fruit, bark and leaf extracts inhibited the proliferation of MDA MB-468 by 95, 94.5 and 85.3%, MCF-7 by 79.8, 9.43 and 49.08%, and Caco-2 by 75.8, 61.8 and 68.62%, respectively. Inhibited DNA synthesis and induced apoptosis of the cancer cells.	Alam et al. (2017)
Methanol extracts of fruits and barks of *Z. heitzii*	10–100 μg/ ml	Human leukemia (HL-60) cells	Cytotoxic against cancer cells with IC_50_ values of 20 and 12 μg/ ml, respectively for fruit and bark extracts. Acted by generating mitochondrial-dependent apoptosis and Go/G1 phase arrest of the cancer cell cycle.	Pieme et al. (2014)
Methanol extract of *Z. heitzii* bark and fruits	1–100 μg/ml	Cervical cancer (HeLa), breast cancer (MCF-7), acute monocytic leukemia (THP-1) and prostate cancer (PC-3) cells	Bark extract showed antiproliferative effects with IC_50_ values of 66, 76, 8.4, and 42 μg/ ml, respectively while the fruit extract was only active against PC-3 and MCF-7 cells with IC_50_ values of 56 and 26 μg/ ml, respectively.	Dzoyem et al. (2013)
Methanol extract of *Z. leprieuii* fruits	1–100 mg/ ml	PC-3, MCF-7, liver (WRL-68), and colon (Caco-2) cells	Cytotoxic against the cancer cells with IC_50_ values of 88, >200, 17 and 60 μg/ml, respectively relative to doxorubicin with IC_50_ values of 5.0, 2.1, 0.85 and 3.3 μg/ml, respectively	Misra et al. (2013)
Methanol extract of *Z. zanthoxyloides* fruits	1–100 mg/ ml	PC-3, MCF-7, WRL-68, and Caco-2 cells	Antiproliferative against the cancer cells with IC_50_ values of 59, 55, 17 and 66 μg/ ml, respectively	Misra et al. (2013)
Dichloromethane/methanol extract of *Z. paracanthum* root bark	0.14–100 μg/ ml	Human breast cancer (HCC 1395) and human prostate cancer (DU 145) cells	Cytotoxic against cancer cells with IC_50_ values of 28.28 and 7.27 μg/ ml, respectively	Kaigongi et al. (2020)

Several studies have isolated the *Z.* species phytochemicals that may be responsible for their *in vitro* anticancer properties. Based on their classes, chemical compounds derived from *Z.* species with substantial inhibitory activities against cultured cancer cells are discussed below. An orbitide, [1-8-NαC]-zanriorb A1, isolated from *Z. riedelianum* Engl. leaves inhibited the proliferation of Jurkat leukemia T cells (IC_50_ 218 nM) by inducing apoptosis (Beirigo et al., 2016). In addition, phenolic compounds isolated from *Z. ailanthoides* stem, chlorogenic acid, flavone and isoflaxidin, were shown to suppress Colo 205 viability and induce apoptosis and cell cycle arrest at the G2/M-phase via upregulation of the expression of apoptosis-inducing factor, endonuclease G, and caspases 3, 7, and 9 while suppressing fatty acid synthase (FAS) (Chou et al., 2011). FAS is a multifunctional enzyme complex that is gaining attention as a target for cancer management. The inhibition of FAS activity in many cancer cells induce restimulation-induced cell death, one of the notable apoptotic pathways (Fhu and Ali 2020). Similarly, an alkamide, 4-(isoprenyloxy)-3-methoxy-3,4-deoxymethylenedioxyfagaramide, isolated from *Z. chalybeum* stem bark was moderately cytotoxic against CCRF-CEM and CEM/ADR5000 cells with IC_50_ values of 29.13 and 31 μM, respectively (Omosa et al., 2019), although the bioactivity mechanism is unknown. Such mechanistic information may facilitate the identification of specific molecular targets and derivatives of the compound with enhanced potency.

Furthermore, *Z.* species have been reported to contain several coumarins with broad-spectrum anticancer activities. For example, coumarins from *Z. schinifolium* stem, collinin, 8-methoxyanisocoumarin H and acetoxyschinifolin, significantly halted the proliferation of PC-3, HL-60, and colorectal (SNUC5) cancer cells with respective IC_50_ values of 4.62, 4.39 and 6.26 μM by collinin, IC_50_ of 5.02, 12.22 and 33.5 by 8-methoxyanisocoumarin H, and IC_50_ values of 5.12, 33.81 and 35.11 μM by acetoxyschinifolin. The coumarins acted by inducing apoptosis and suppression of the expression of genes (p-ERK1/2 MAPK, p-AKT, and c-myc) involved in cancer development and progression (Li et al., 2013). Furthermore, a pyranocoumarin from *Z. ailanthoides* stem bark, luvangetin exhibited weaker cytotoxic activity against human lung cancer (A-549) cells with an IC_50_ value of 4.28 μg/ ml compared to 5-fluorouracil, a known anticancer agent (IC_50_ of 0.6 μg/ ml) (Cao et al., 2013). Nonetheless, the potential of luvangetin can be further explored by structural modification to possibly obtain more potent anticancer derivatives.

*Zanthoxylum-*isolated lignans have also been reported to have anticancer activities. Sesamin from *Z. parachanthum* demonstrated cytotoxic activity against CCRF-CEM and CEM/ADR5000 cancer cells with IC_50_ values of 40.74 and 30.70 µM, respectively (Omosa et al., 2019). In addition, (-)-xanthoxylol-3,3-dimethylallyl ether from *Z.* bungeanum Maxim stem bark was cytotoxic against MCF-7 cancer cells with an IC_50_ of 18.65 μg/ ml (Yang et al., 2009) while asarinin from *Z. americanum* stem suppressed the proliferation of HL-60 cancer cells with IC_50_ of 11.64 μM (Ju Y et al., 2001). Interestingly, the cytotoxicity of kobusin from *Z. rhetsa* bark against mouse melanoma (B16-F10) cells was weaker (IC_50_ values of 112.2 μg/ ml) Santhanam et al. (2016) than the activity of kobusin from *Z. armatum* bark against human lung (A549) and pancreatic (MIA-PaCa) cancer cells (IC_50_ values of 34.71 and 32.86 μg/ ml, respectively) (Mukhija et al., 2014). This demonstrates that activity is dependent on the cancer cell type, possibly because of differences in bioaccessibility of the compounds, their molecular targets and anticancer mechanisms. In general, the lignans acted by inducing apoptosis and cell cycle arrest, and inhibiting DNA synthesis in the cancer cells. In addition to the broad-spectrum antiproliferative properties, the potential of these lignans as anticancer agents is strengthened by the absence of cytotoxicity to human dermal fibroblasts and peripheral blood mononuclear cells.

In many studies, *Zanthoxylum* alkaloids were reported to exhibit cytotoxic activity against cancer cells *in vitro* (Wei et al., 2021). Specifically, a furoquinoline alkaloid, skimmianine, demonstrated cytotoxic activity against MCF-7 cancer cells with an IC_50_ value of 8.03 μg/ ml while an aporphine alkaloid, liriodenine, was cytotoxic against MCF-7, NCI-H460, and SF-268 cancer cells with IC_50_ values of 3.19, 2.38 and 2.19 μg/ ml, respectively (Andima et al., 2020). Recently, acridone alkaloids, fabiocinine and arborinine, and skimmianine from *Z. leprieu*rii Guill. and Perr. root bark were reported to exhibit selective cytotoxicity against HeLa cells with IC_50_ values of 28.49, 62.71 and 12.8 μg/ ml, respectively, which were lower than the activity of anticancer agent, emetine (IC_50_ values of 0.026 μg/ ml against HeLa cells) (Eze et al., 2020). Similarly, an indole alkaloid, canthin-6-one from *Z. parachanthum* inhibited CCRF-CEM and CEM/ADR5000 cancer cell proliferation with IC_50_ values of 15.82 and 10.52 µM, respectively (Omosa et al., 2019). Unlike doxorubicin, canthin-6-one demonstrated selective cytotoxicity against the drug-resistant cell line without affecting normal human peripheral blood mononuclear cells (Omosa et al., 2019). In other cancer cells (HCC 1395 and DU 145), canthin-6-one and its derivative, 10-methoxycanthin-6-one, from the same plant were strongly cytotoxic with IC_50_ values of 8.12, and 9.43 μg/ ml, and 14.70 and 1.58 μg/ ml, respectively (Kaigongi et al., 2020). Despite its lower cytotoxicity against cancer cells compared to doxorubicin, the better selectivity/nontoxicity to normal cells positions canthin-6-one as a promising candidate with a broad-spectrum anticancer activity.

Furthermore, benzophenanthridine alkaloid, 1-methoxy-12-methyl-12,13-dihydro-(1,3) dioxolo (4′,5′:4,5) benzo (1,2-c) phenanthridine-2,13-diol, from the aerial parts of *Z. buesgenii* (Engl.) P.G.Waterman showed moderate to strong cytotoxicity against sensitive and multidrug resistant cancer cells (CCRF-CEM, CEM/ADR5000, MDA-MB231, MDA-MB231/BCRP, HCT116 (p53^+/+^), HCT116 (p53^−/−^), U87MG, U87MG.ΔEGFR, and HepG2) with IC_50_ values of 0.24, 31.58, 30.14, 65.01, 42.46, 62.34, 60.55, 61.84, and 22.37, respectively, while sparing normal human liver (AML12) cells (Sandjo et al., 2014). Due to the broad-spectrum anticancer activity of the benzophenanthridine and aporphine alkaloids, further studies are required to understand the molecular mechanism of action against the cancer cells. Benzophenanthridine alkaloid from another species (Z. madagascariense Baker), rutaceline, showed inhibitory activity against Caco-2 cells by inducing apoptosis, cell cycle arrest at the G0/G1 phase and DNA fragmentation, and by inhibiting DNA synthesis (Pachón et al., 2007). Acting via similar mechanisms (induction of apoptosis and cell cycle arrest by strong binding to cyclin-dependent kinases (CDK2 and CDK6) and caspases 3 and 8), the ability of a benzophenanthridine alkaloid from *Z. zanthoxyloides* roots, dihydrochelerythrine to exhibit significant cytotoxicity against HCC and BT549 cancer cells Andima et al. (2020) demonstrates the strong anticancer potential of the benzophenanthridine alkaloids from *Z.* species. Through unknown mechanisms, other alkaloids such as isoquinoline alkaloids (e.g. nitidine, fagaronine chelerythridine and sanguinarine) from *Z. bungeanum* elicited selective DNA damage and cytotoxicity against mouse lymphocytic leukemia cells *in vitro* (Kaminskyy et al., 2008; Liao et al., 2013; Tian et al., 2017). Similarly, *Z. austrosinense* C.C. Huang root-derived carbazole alkaloids, zanthoaustrones A–C, exhibited strong antiproliferative activities against human leukemia (HL-60), liver (SMMC-7721), lung (A-549), breast (MCF-7) and colon (SW480) cancer cell lines (Fu et al., 2020). Despite the promising *in vitro* anticancer activities reported, the experimental designs often did not include appropriate positive controls. This is needed for validation of anticancer activities prior to *in vivo* studies, considering potential differences in assay conditions that may influence cellular activities.

Redox imbalance in cancer cells caused by reduction in antioxidant status and elevation of ROS production and lipid peroxidation has been targeted as a major mechanism through which some plant-derived compounds induce apoptosis (Redza-Dutordoir and Averill-Bates 2016). Other mechanisms include caspase-mediated signaling, which induces apoptosis, and p53-mediated cell cycle arrest (Zhang Y. et al., 2017). For example, an acridone alkaloid derivative (2-aminoacetamido-10-(3,5-dimethoxy)-benzyl-9(10H)-acridone hydrochloride) was shown to kill leukemia cells by decreasing mitochondrial transmembrane potential while increasing the expression of Bax, cytochrome C and apoptosis protein activating factor-1 to form an apoptosome (Wang et al., 2013). Formation of apoptosome activates caspase-9 with concomitant activation of caspase-3, the final inducer of apoptosis. The increase in intracellular ROS production induced by natural products also alters membrane phospholipid composition and integrity, all of which contribute to cancer cell death (Rahman et al., 2021).

Taken together, *Z.* species contain a repertoire of phytochemicals with promising application in the treatment of cancer. However, the reviewed studies were conducted in cell cultures *in vitro*, without validation of physiological anticancer effects of the extracts or isolated compounds using model organisms or in humans. This is a major limitation of the studies because of pharmacokinetic and pharmacodymanic considerations, which influence the bioaccessibility, bioavailability and target binding/sensitivity of the compounds. Furthermore, some cancer cells have developed mechanisms for resisting the cytotoxic actions of some anticancer agents, such as reduced expression of drug targets while upregulating the expression of alternative survival pathways (Pistritto et al., 2016; Bukowski et al., 2020). Consequently, the multiple target mechanisms of anticancer activities identified *in vitro* for some *Z.* species-derived compounds make them strong candidates for *in vivo* studies and human clinical trials, and further development as anticancer agents. Chemical compounds isolated from *Z.* species with anticancer properties and their molecular mechanisms are presented in Table 2; Figure 1.

**TABLE 2:** Chemical compounds isolated from *Zanthoxylum* species with *in vitro* anticancer properties.

Compounds	Class of compound	*Z.* species of source	Cancer cell model	Mechanism of action	Reference
[1-8-NαC]-zanriorb A1	Orbitide	*Z. riedelianum* leaves	Jurkat leukemia T cells	Induced apoptosis	Beirigo et al. (2016)
Chlorogenic acid	Phenolics	*Z. ailanthoides* stem	Human colon cancers (Colo 205)	Induced apoptosis and cell cycle arrest at the G2/M-phase	Chou et al. (2011)
Flavone					
Isoflaxidin					
4-(isoprenyloxy)-3-methoxy-3,4-deoxymethylenedioxyfagaramide	Alkamide	*Z. chalybeum* stem bark	CCRF-CEM and CEM/ADR5000 cancer cells	NR	Omosa et al. (2019)
Collinin	Coumarin	*Z. schinifolium* stem	PC-3, HL-60 and colorectal (SNUC5) cells	Induced apoptosis and suppressed the expression of p-ERK1/2 MAPK, p-AKT, and c-myc genes	Li et al. (2013)
8-methoxyanisocoumarin H					
Acetoxyschinifolin					
Luvangetin	Coumarin	*Z. ailanthoides* stem bark	A-549 cells	NR	Cao et al. (2013)
Sesamin	Lignan	*Z. parachanthum*	CCRF-CEM and CEM/ADR5000 cells	NR	Omosa et al. (2019)
(-)- xanthoxylol-3,3-dimethylallyl ether	Lignan	*Z. nitidum* stem bark	MCF-7 cells	NR	Yang et al. (2009)
Asarinin	Lignan	*Z. americanum* stem	HL-60 cancer cells	NR	Ju Y et al. (2001)
Kobusin	Lignan	*Z. armatum* bark and *Z. rhetsa* bark	mouse melanoma (B16-F10) cells	NR	Mukhija et al., 2014; Santhanam et al., 2016
Skimmianine	Alkaloid	*Z. bungeanum* root	MCF-7 cells	NR	Andima et al. (2020)
Liriodenine	Alkaloid	*Z. zanthoxyloides* root	MCF-7, NCI-H460, and SF-268 cancer cells	NR	Andima et al. (2020)
1-methoxy-12-methyl-12,13-dihydro-[1,3]dioxolo [4′,5′:4,5]benzo [1,2-c]phenanthridine-2,13-diol	Alkaloid	*Z. buesgenii* aerial parts	CCRF-CEM, CEM/ADR5000, MDA-MB231, MDA-MB231/BCRP, HCT116 (p53^+/+^), HCT116 (p53^−/−^), U87MG, U87MG.ΔEGFR, and HepG2 cells	NR	Sandjo et al. (2014)
Rutaceline	Alkaloid	*Z. madagascariense* bark	Caco-2 cells	NR	Pachón et al. (2007)
Dihydrochelerythrine	Alkaloid	*Z. zanthoxyloides* roots	HCC and BT549 cells	NR	Andima et al. (2020)
Nitidine	Alkaloid	*Z. bungeanum* stem	Mouse lymphocytic leukemia cells	Induced apoptosis and DNA damage	Liao et al., 2013; Tian et al., 2017
Fagaronine					
Chelerythridine					
Sanguinarine					
Zanthoaustrones A–C	Alkaloid	*Z. austrosinense* root	HL-60 cells	NR	Fu et al. (2020)
Canthin-6-one	Alkaloid	*Z. parachanthum* bark	CCRF-CEM, CEM/ADR5000, HCC 1395 and DU 145 cells	NR	Omosa et al., 2019; Kaigongi et al., 2020
10-methoxycanthin-6-one	Alkaloid	*Z. parachanthum* bark	HCC 1395 and DU 145 cells	NR	Kaigongi et al. (2020)
hyperoside	flavonol glycoside	*Z. bungeanum* leaves	Human colorectal cancer cells (SW620)	Induced cell cycle arrest at G2/M phase and apoptosis	Zhang et al. (2017b)

NR, Not reported.

## *Zanthoxylum* Species as Potential Sources of Antimicrobial Agents

Microorganisms play many roles essential for human survival and are used as sources of drugs such as antibiotics. However, many strains of bacteria and fungi, such as *Streptococcus mutans*, *S. aereus*, *Mycobacterium tuberculosis*, *K. pneumoniae, Candida* species*,* and *Escherichia coli* are causative agents of many diseases of clinical importance. Several classes of antimicrobial drugs are used to control microbial infections, by suppressing microbial growth or killing them. However, survival pressure has led to the emergence and spread of antibiotics-resistant strains of many microorganisms, including those that are resistant to multiple drugs of the same or different classes (multi-drug resistant strains). These drug resistant strains have led to prolonged treatment duration, frequent hospitalization, increased healthcare cost and mortality from treatable microbial infections (Naylor et al., 2018). This necessitates the urgent search for clinically effective antimicrobial agents against these “superbugs”.

Antimicrobial agents from herbs that are traditionally used in treating microbial infections are being isolated and assessed for activities against drug-resistant microbial strains. Many natural products derived from genus *Zanthoxylum* show promising antimicrobial activities against bacteria and fungi of public health importance. Investigations for antimicrobial activities are mostly guided by traditional uses of the plant species in the treatment of infectious diseases. Among the *Z.* species, *Z. zanthoxyloides* is well known for its use in the management of microbial infections in China and Korea and other parts of Asia as well as in Uganda, Nigeria and Ghana (Anokbonggo et al., 1990; Ngono-Ngane et al., 2000; Imaga 2010; Ynalvez et al., 2012; Ouédraogo et al., 2019; Adeeyo et al., 2020). Additionally, *Z. zanthoxyloides* is used as chewing stick and to treat oral infections and toothaches (Anyanwu and Okoye 2017), thus suggesting that the plant species may have antimicrobial activities against oral pathogens. Similarly, *Z. rhetsa* has been used against urogenital microbial infections and in disinfection of contaminated surfaces in Bangladesh (Yusuf et al., 1994), while *Z. lemairei* (De Wild) P.G. Waterman is used against malaria and diarrhea (Adesina et al., 1997), *Z. chalybeum* stem bark against malaria in Rwanda and Ivory Coast (Kamanzi Atindehou et al., 2002), and *Z.* schreberi (J.F.Gmel.) Reynel ex C. Nelson in treating eye infections in the Caribbean, Venezuela, Colombia and Costa Rica (Rodríguez-Guzmán et al., 2011).

Instead of the whole plant parts, solvent extracts of different parts of *Z.* species have been investigated for antimicrobial activities. As shown in Table 3, the potency of antimicrobial activity [expressed as minimum inhibitory concentration (MIC) or inhibition zone diameter (IZD)] varies with the different plant species, part used (fruits, leaves, root bark, stem bark), microorganism tested, solvent used for extraction, or type of assay used in the studies. Moderately polar solvents appear to be the best medium for extraction of the antimicrobial compounds compared with highly polar and non-polar solvents (Gonçalves et al., 2019). It is also worth noting that most of the microorganisms studied are drug-sensitive species and only a few studies tested the extracts on drug-resistant microorganisms. Thus, it is challenging to assess and conclude on the potential of individual plant extracts as presented in the literature. It is apparent that the *Z.* species crude extracts contain antimicrobial principles, which may need to be purified to enhance the activity or for elucidating molecular mechanisms. Nonetheless, the combination of several potentially active principles in the crude extracts may present an opportunity. For instance, aqueous-methanol extract of *Z. zanthoxyloides* root bark showed antibacterial activity against *Streptococcus mutans, Sarcina lutea,* and *Lactobacillus* sp. at IZD of 20, 32, and 56 mm, respectively, at 100 mg/ ml compared to the stronger but limited effect of antibiotic drug, amoxicillin, which was active (IZD of 22 mm at 10 μg/ ml) only against *Lactobacillus* sp. (Okafor et al*.,* 2017). Multi-component extracts with such promising antimicrobial activity can be further investigated for safety and pharmacological effects as low-cost alternatives to purified compounds or drugs. Aside from solvent extracts, the antimicrobial activities of multi-component essential oils derived from *Z.* species have been reported (Table 3). In one study, bioautophagy-directed fractionation of essential oil from Z. armatum leaves led to the isolation of β-fenchol and linalool, which had antifungal activities against *A. alternata* and *C. lunata* (Guleria et al., 2013).

**TABLE 3:** Summary of the antimicrobial activities of solvent extracts and essential oils from different part of *Zanthoxylum* species.

Plant species and part used	Test substance	Microorganism targeted	Activity	References
*Z. zanthoxyloides* fruits	Crude methanol extract	*P. aeruginosa*	IZD of 15 mm	Misra et al. (2013)
	Essential oil	*K. pneumonia*, *P. aeruginosa* and *S. typhimurium*.	IZD of 12, 11, and 9 mm, resp.	
*Z. leprieurii* fruits	Crude methanol extract	*P. aeruginosa*	IZD of 15 mm	Misra et al. (2013)
*Z. zanthoxyloides* root bark	Aqueous-methanol extract	*S. mutans, S. lutea, C. albicans,* and *A. niger*	IZD of 20–32 mm	Okafor et al*.* (2017)
*Z. zanthoxyloides* fruits	Essential oil	*S. aureus, E. coli, E. faecalis,* and *C. albicans*	IZD of 8.6–18.8 mm	Tine et al. (2017)
*Z. leprieurii* root bark	Methanol extract	Pan sensitive (H37rv), rifampicin resistant (TMC 331) and isoniazid resistant (TMC 301) strains of *M. tuberculosis*	MIC of 47.3, 75.3 and 125 μg/ ml	Bunalema et al. (2017)
*Z. zanthoxyloides*	Ethylacetate and chloroform extracts	*E. coli, P. aeruginosa, Klebsiella sp, S. pneumoniae* and *B. cereus*) and fungal species (*A. niger, A. flavus, Trichoderma sp* and *Candida sp*.	IZD of 7.5–16 mm at 25 mg/ ml	Adeeyo et al. (2020)
*F. heitzii* fruits and root bark	Ethanol extract	*E.coli, P. aeruginosa, C. albicans,* and *A. fumigatis*	MIC of 500 μg/ ml (fruit) and 1,000 μg/ ml (root; for only *A. fumigates*)	Dzoyem et al. (2013)
*Z. clava-herculis* leaves and stem bark	Methanol extract	*E. coli* (AG102), *E. aerogenes* (EA27), *K. pneumonia* (KP63) and *Providencia stuartii* (NEA16)	MIC of 64–512 μg/ ml	Seukep et al. (2015)
*Z. bungeanum* fruits	Essential oil	*E. coli*	MIC of 24 mg/ ml (cell wall lysis *in vitro*)	Hong et al. (2017)
*Z. chalybeum* stem bark	Dichloromethane and ethanol extracts	*S. aureus, S. typhi* and *P. aeruginosa*	Dichloromethane extract, MIC of 32 μg/ml (*S. aureus*); ethanol extract, MIC values of 32, 250 and 500 μg/ml (*S. aureus, S. typhi* and *P. aeruginosa*, resp.)	Mugiraneza et al. (2013)
*Z. chalybeum* stem bark	Dichloromethane, ethylacetate and methanol extracts	Isoniazid-resistant strains of *M. madagascariense* and *M. indicus pranii*	Dichloromethane extract, MIC of 1.25 mg/ ml (both); methanol extract, MIC of 1.25 and 2.5 mg/ ml, resp.	Chrian et al. (2011)
*Z. ovalifolium* fruit	Hexane, ethylacetate and methanol extracts	*K. pneumonia* and *S. aureus*	Ethyl acetate extract, IZD of 15 and 16 mm, resp.; n-hexane extract, IZD of 14 and 10 mm, resp.; methanol extract, IZD of 13 and 14 mm, resp. at 100 μg/ ml	Pavani and Naika (2020)
*Z. paracanthum* root bark	Chloroform-ethanol extract	Methicillin-resistant *S. aureus* (MRSA), *E. coli* (ATCC 25922), *S. aureus* (ATCC 29213) and *C. albicans* (ATCC 10231)	MIC of 3.91, 0.98, 1.95 and 7.81 μg/ml, resp.	Kaigongi et al. (2020)
*Z. bungeanum* leaves	Essential oil	*S. aureus, B. cereus, B. subtilis*, *L. monocytogenes*, *S. choleraesuis, V. parahaemolyticus, A. hydrophila, S. sonnei, V. vulnificus* and *S. enterica*	MIC of 1.25 μg/ ml	Lee et al. (2012)
*Z. tingoassuiba* roots	Methanol and dichloromethane extracts	*S. aureus* ATCC 25923 and four multidrug resistant strains of the bacterium	Methonol extract, IZD of 18.3–23.3; dichloromethane extract, IZD of 13.3–20.3 mm	Costa et al. (2017)
*Z. armatum* leaves	Crude methanol extract and essential oil	*Alternaria alternata*, and *Curvularia lunata*.	MIC of 1,071 and 948 μg/ ml, resp.	Guleria et al. (2013)
*Z. leprieurii* fruits	Methanol extract	*S. aereus and S. saprophyticus*	MIC of 2 and 7 μg/ ml, resp.	Njimoh et al., 2015
*Z. acanthopodium* fruits		*M. smegmatis*	MIC value of 64 μg/ ml	Julistiono et al. (2018)
*Z. armatum* seeds and fruits	Crude methanol extract	*S. aureus, B. subtilis, E. faecalis,* MRSA, and *S. epidermidis*	IZD of 11.73–20.72 mm	Phuyal et al. (2020)

IZD, inhibition zone diameter; MIC, minimum inhibitory concentration.

In general, studies on the *Z.* species crude extracts and essential oils reported antimicrobial activity as IZD or MIC, and seldom investigated their molecular mechanisms. In one study, n-hexane extract of *Z. acanthopodium* fruits that was active against *M. smegmatis* was reported to induce loss of intracellular sodium and potassium ion concentration, suggesting that the extract acted by damaging the bacterial cell wall (Julistiono et al., 2018). Hong et al. (2017) also reported that essential oil from *Z. bungeanum* fruits, containing 6,9,12,15-hexadeca-tetraenoic acid-methyl ester, 4-terpinenylacetate, D-limonene, eucalyptol, α-terpineol, β-linalool, δ-cadinene and β-pinene, caused lysis of cultured *E. coli* membrane. This mechanism was supported by the high amount of bacterial intracellular (nucleic acids and proteins) and cell membrane components in the culture medium. *In vivo* evaluation in a mouse model of enteritis demonstrated that *Z. bungeanum* fruit-derived essential oil downregulated the expression of pro-inflammatory cytokines (Hong et al., 2017); this indicates that anti-inflammatory mechanism played a role in host protection by the essential oil against *E. coli* infection. Indeed, elucidation of molecular mechanisms would be more logical for isolated compounds with defined molecular targets in the microorganisms or host. Nonetheless, knowledge of the molecular basis of antimicrobial effect would enhance the direct utilization of crude extracts of *Z.* species for pharmacological applications.

Efforts have been made to isolate compounds that are responsible for the reported antimicrobial activities of *Z.* species extracts. Most of the compounds are alkaloids and some possess antimicrobial activity against both drug-sensitive and drug-resistant species of public health importance. For example, alkaloids (6-acetonyldihydronitidine, 6-acetonyldihydroavicine and 6-acetonyldihydrochelerythrine) from the stem bark of *Z. rhoifolium* strongly inhibited the growth of *S. aureus, S. epidermidis, K. pneumonie, S. Setubal,* and *E. coli* with respective MIC values of 1.06, 1.06, 3.12, 3.12, and 1.06 μg/ ml (6-acetonyldihydronitidine), 1.06, 3.12, 1.06, 3.12, and 3.12 μg/ ml (6-acetonyldihydroavicine), and 12.5, 6.25, 6.25, 6.25, and 12.5 μg/ ml (6-acetonyldihydrochelerythrine) (Gonzaga et al., 2003)*.* Other antimicrobial alkaloids isolated from *Z.* species include dihydrochelerythrine from *Z. rhetsa* roots and stem (Tantapakul et al., 2012); *bis*-[6-(5,6-dihydro-chelerythrinyl)] ether, 6-ethoxy-chelerythrine and 4-methoxy-N-methyl-2-quinolone from *Z.* schreberi leaves and bark (Rodríguez-Guzmán et al., 2011); β-carboline alkaloids (10-methoxycanthin-6-one and canthin-6-one) and phenanthridine alkaloids (8-acetonyldihydrochelerythrine and 8-oxochelerythrine) from *Z. paracanthum* root bark (Kaigongi et al., 2020); N-methylcanadine from *Z. tingoassuiba* A. St.-Hil. roots (Costa et al., 2017); and acridone alkaloids (hydroxy-1,3-dimethoxy-10-methyl-9-acridone and 3-hydroxy-1,5,6-trimethoxy-9-acridone) from *Z. leprieurii* stem bark (Bunalema et al., 2017). Similar to its broad-spectrum anticancer activity, canthin-6-one was strongly active against several bacteria (*S. aureus, E. coli*, *Proteus vulgaris* and *Klebsiella aerogenes*) and fungi (*A. niger* and *C. albicans*), with corresponding MIC values of 0.227, 0.114, 0.114, 0.227, and 0.114 µM, respectively. Furthermore, the acridone alkaloids strongly inhibited first line drug-resistant (H37rv)*,* rifampicin-resistant (TMC 331) and isoniazid-resistant (TMC 301) strains of *M. tuberculosis* with MIC values of 1.5–8.3 μg/ ml (Bunalema et al., 2017), which positions *Z. leprieurii* as a potential source of anti-tuberculosis agents. Structure-activity relationship studies are needed to identify the pharmacophores of the alkaloids against specific microorganisms or their molecular targets. Likewise, antimicrobial mechanisms of the alkaloids are largely unknown, although Wouatsa et al. (2013) reported that acridone alkaloids, 3-hydroxy-1,5,6-trimethoxy-9-acridone and 2,4′-hydroxyzanthacridone oxide, from *Z. leprieurii* fruit extract acted by inhibition of aromatase and glycosyltransferase, which are involved in biosynthesis of bacterial lipopolysaccharides and cell wall.

**FIGURE 2:**
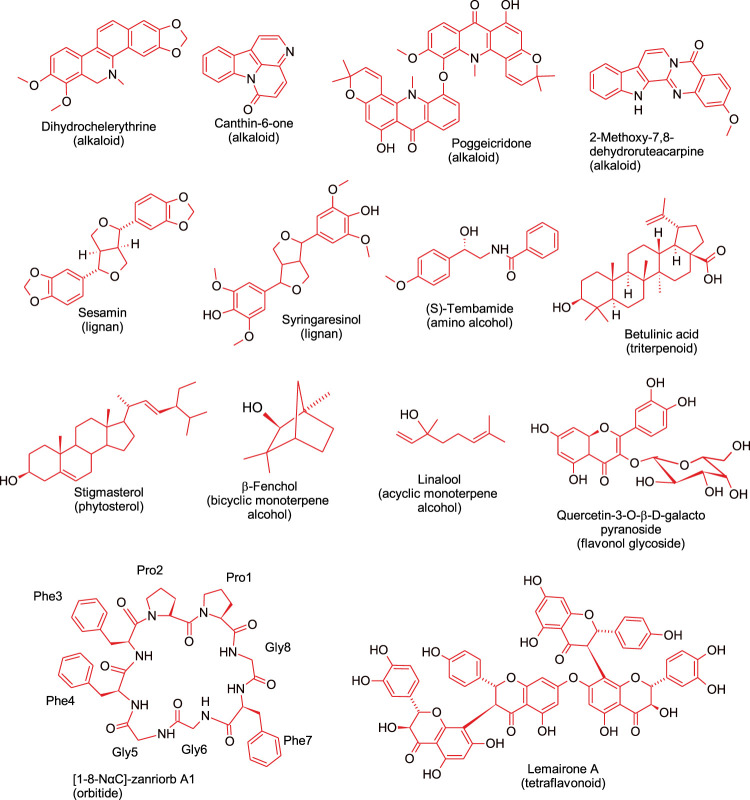
Representative bioactive compounds, from different phytochemical classes, isolated from *Zanthoxylum* species.

Apart from alkaloids, other antimicrobial compounds isolated from different *Z.* species include lignans (e.g., sesamin and syringaresinol) (Rahman et al., 2008), tetraflavonoids (e.g., lemairones A and B) (Bitchagno et al., 2015), and phytosterol (e.g., stigmasterol) (Kaigongi et al., 2020). Lemairones A and B from *Z. lemairei* leaves moderately inhibited multidrug-resistant bacteria, *K pneumoniae* KP55 and *E. coli* AG100 with MIC values of 128 mg/ ml and 64 mg/ ml, respectively against *E. coli* AG100, and MIC value of 128 mg/ ml against *K. pneumoniae* KP55 (Bitchagno et al., 2015). Furthermore, a polymeric procyanidin from *Z. bungeanum* fruit exhibited cytotoxicity against drug resistant strains of *S. aureus* with an MIC value of 128 μg/ ml; the compound acted by inhibiting β-lactamase activity and by inducing cell wall damage (Kusuda et al., 2006). This study also showed that the isolated compound has potential for further development as an adjuvant of antibacterial drugs for mitigating the burden of drug-resistant microbial infections. Taken together, it is recommended that future studies should use drug resistant microorganisms since the ultimate goal is to overcome antimicrobial resistance. Findings from the studies reviewed suggest that some of the compounds isolated from *Z.* species have promising future as source of new antibiotics. Figure 2 shows representative *Z.* species*-*derived compounds with anticancer, antiparasitic, antimicrobial or anti-sickling activities.

## Antiviral Potentials of *Zanthoxylum* Species

The emergence and spread of viruses such as SARS-CoV-2, HIV and hepatitis B have led to heightened efforts in search for effective remedies. These efforts include repurposing of drugs developed for other viral diseases as well as exploring for new drug candidates in medicinal plants used in treating viral infections by traditional medicine practitioners. A few studies have reported antiviral activities for extracts from *Z.* species. Following the folkloric use in treating oral pathogens and symptoms related to picornaviridae infection, Choi (2016) found that the methanol extract of *Z. piperitum* leaves were cytotoxic to human rhinoviruses - HRVs (HRV2 and HRV3) and enteroviruses (coxsackie A16, B3, and B4 viruses, and human enterovirus71) of picornaviridae virus family with IC_50_ values of 59, 39, 45, 68, 93, and 4.4 μg/ml, respectively. As the extract had low toxicity against human cells (Hela and Vero cells), the active ingredients, if isolated, can serve as bioactive candidates against viral diseases caused by members of the picornaviridae family. Moreover, leaves of *Z. bungeanum* are used in Korea and Japan for treating respiratory diseases. To support this use, Ha et al. (2014) reported the anti-influenza virus A/NWS/33 (H1N1) effects of flavonol glycosides, quercetin-3-O-β-D-galactopyranoside, quercetin-3-O-α-L-rhamnopyranoside and kaempferol-3-O-α-L-rhamnopyranoside isolated from the *Z. bungeanum* leaves; the flavonol glycosides also inhibited influenza A virus neuraminidase activity with IC_50_ values of 434, 211, and 273 μg/ ml, respectively. Influenza A virus neuraminidase is involved in the release of newly made virus particle from infected cells, making it a good target for reducing the spread in host cells. Considering the multiple molecular targets of polyphenols, it is also possible that the antiviral activity was mediated via additional unknown mechanisms.

In addition to influenza virus, the antiviral potentials of *Z.* species-derived phytochemicals have also been reported against hepatitis B virus. A coumarin, collinin, from chloroform extract of *Z. schionifolium* bark exhibited anti-hepatitis B virus activity (ED_50_ of 68.3 μg/ ml) and also inhibited HBV-DNA replication (IC_50_ of 17.1 μg/ ml) (Chang et al., 1997). Using a different assay, two alkaloids, 5,6-dihydro-6-methoxynitidine and 5-methoxydictamnine, from Z. bungeanum Maxim roots at 0.2 µM showed higher anti-HBV activities by respectively inhibiting 43.3 and 49.3% of viral multiplication than 10 µM of standard antiviral drug, lamivudine with 29.6% inhibition (Yang and Chen 2008). In addition, a benzophenanthridine alkaloid (decarine), a furoquinoline alkaloid (γ-fagarine), and an amino alcohol derivative ( + )-tembamide) from Z*. ailanthoides* root bark showed anti-HIV activities (EC_50_ values <0.05 μg/ ml) with no cytotoxicity against normal H9 lymphocyte cells (Cheng et al., 2005). It is challenging to compare the antiviral potential of the *Zanthoxylum* compounds because of differences in the structural type of the compounds, target virus, and assay method used in these studies. Nonetheless, due to their selected toxicity to viruses, the *Z.* species*-*derived alkaloids warrant further investigation for potential use in treating viral infections.

## *Zanthoxylum* Species as Potential Source of Anti-parasitic Agents

Human African trypanosomiasis (sleeping sickness), a neglected tropical disease, is a *Trypanosoma* species-caused parasitic disease that is endemic in sub-Saharan Africa, where majority of the victims are poor rural dwellers. *Trypanosoma* species is transmitted by tsetse flies and cause a fatal disease if not properly treated. A few *T.* species*,* such as *T. brucei gambiense,* is responsible for the vast majority of *Trypanosoma* infection in West and Central Africa while *T. rhodesiense* is mostly responsible for infections in East Africa where less than 10% of the infection exists. The initial (hematolymphatic) stage of infection is asymptomatic and, if detected early, is treatable with pentamidine or suramin while the second (meningo-encephalitic) stage, which is characterized by the invasion of the central nervous system by the parasite, is treatable with melarsoprol or eflornithine (Simarro et al., 2011). Unfortunately, these drugs do not guarantee total recovery as the success rate in most cases is less than 90%. In addition to the growing drug resistance of the parasites, some devastating toxicities accompy the use of these drugs (Shaw et al., 2010). This presents the need for safer and potent alternatives, especially from natural products and medicinal plants that have a history of application in treating the disease. Medicinal plants are used for treating trypanosomiasis in Nigeria (Atawodi et al., 2003; Bulus et al., 2016; Okwor et al., 2020); thus, they have strong prospects for use as sources of clinically relevant anti-trypanosoma agents. In Nigeria and Ghana, roots, stem and leaves of *Z. zanthoxyloides* are used in treating trypanosomiasis (Mann et al., 2011). When cultured with the root extract, the viability of *T. brucei* was demonstrated to be suppressed (IC_50_ = 3.41 μg/ ml) by induction of apoptosis and cell cycle arrest at G0/G1 phase (Dofuor et al., 2019). Subsequent investigation by the same group resulted in the isolation of an alkaloid, skimmianine, and an oxylipin, 9-oxo-10,12-octadecadienoic acid, from *Z. zanthoxyloides* root as the cytotoxic principles against *T. brucei* (GUTat 3.1 strains; EC_50_ values of 1.7 and 1.2 µM, respectively) (Dofuor et al., 2020). Although less active than diminazene aceturate, an antitrypanosomal drug (EC_50_ of 0.5 µM), the alkaloids acted by inducing cell cycle arrest at G0-G1 and (G2-M) phases and by inhibiting DNA synthesis of the parasite. Similarly, an acridone alkaloid, arborinine, derived from *Z. leprieurii* stem bark exhibited anti-trypanosomal activity in cultured *Trypanosoma brucei* (s427) cells with an IC_50_ of 13.2 μg/ ml by unknown mechanisms (Eze et al., 2020). There is a need to test the clinical effectiveness of these isolated compounds in trypanosoma infection to clarify if their *in vitro* activities can translate into clinically relevant *in vivo* effects. This is because some therapeutic agents that are active in culture studies are not biostable in the gastrointestinal tract or may face transport barriers during transepithelial transport when orally ingested (Udenigwe et al., 2021).

Apart from trypanosomiasis, malaria is another parasitic disease targeted with some *Z.* species. Malaria is caused by *Plasmodium* species and is transmitted by *Anopheles* species through the blood of an infected human. An estimate of over 200 million people die from malaria-related events and a vast majority of these deaths occur in sub-Saharan Africa with Nigeria bearing the highest burden (WHO, 2019). Majority of those who contract and die from malaria are poor rural dwellers who resort to cheap and ineffective drugs that relieve the symptoms, leading to relapses and increase in the development of resistant strains of the parasite (Karunamoorthi et al., 2013). In addition, some individuals are sensitive to some prescription antimalarial drugs (Haakenstad et al., 2019). Consequently, traditional medicine practitioners harness the therapeutic potentials of medicinal plants to treat malaria. Many *Zanthoxylum* species have been investigated as sources of antimalarial agents. For example, an *in vitro* study by Mofor et al. (2017) reported that extract of *Z. clava-herculis* stem bark inhibited multidrug resistant strain of *P.* species with IC_50_ of 4.94 μg/ ml and with low toxicity against monkey kidney epithelial cell line. Despite the prospects, the phytochemicals responsible for the antimalarial activity and their mechanism of action are unknown. Other studies have attempted to isolate some antimalarial principles from *Z.* species. Among the compounds, alkaloids, lignans and amides dominated as active compounds. For instance, sesamine from *Z. gilletii* stem bark showed significant anti-plasmodial activities against chloroquine-sensitive Sierra Leone (D6), chloroquine-resistant Indochina (W2), and artemisinin-resistant strain (3D7) of *P. falciparum* with IC_50_ of 1.92, 3.23, and 2.94 μg/ ml, respectively (Masinde 2014). Secondary metabolites such as syncarpamide and decarine from *Z. syncarpum* stem also significantly inhibited both chloroquine-sensitive and chloroquine-resistant strains of malaria parasite; IC_50_ values of 2.04 and 1.44 µM were recorded against *P. falciparum* D6 strain and 3.06 and 0.88 µM against *P. falciparum* W2 strain (Ross et al., 2004). Syncarpamide was cytotoxic against African green monkey kidney (VERO) fibroblast cell line only at high concentration of 56 μM, outside the range of bioactivity concentrations. This suggests that the *Z. syncarpum* compounds can potentially exhibit anti-plasmodial effect with low toxicity to the host.

Based on the use of different *Z. zanthoxyloides* parts for treating malaria (Adesina 2005; Enechi et al., 2019), Goodman et al. (2019) that four alkaloids, bis-dihydrochelerythrinyl ether, skimmianine, buesgenine and chelerythrine, isolated from roots, root-bark and stem-bark exhibited anti-plasmodial activity against chloroquine-sensitive (3D7) strains of *P. falciparum* (IC_50_ values of 4.3, 0.7, 2.0, and 0.4 μg/ ml, respectively). Previous studies on other alkaloids showed that nitidine from *Z. gilletii* stem bark exhibited anti-plasmodial activity against *P. falciparum* strain FcB1 with IC_50_ < 5 μg/ml by halting DNA synthesis in the parasite (Zirihi et al., 2005; Zirihi et al., 2009). Moreover, 8-acetonyldihydrochelerythrine from *Z. gilletii* stem bark inhibited D6, W2, and 3D7 strains of *P. falciparum* with IC_50_ values of 4.06, 4.02, and 3.37 μg/ ml, respectively through unknown mechanisms (Masinde, 2014). Apart from alkaloids, an amide, fagaramide, isolated from *Z. gilletii* stem bark was moderately active (IC_50_ of 7.73, 15.15, and 7.72 μg/ ml) against D6, W2, and 3D7 strains of *P. falciparum*, respectively (Masinde, 2014). Another amide, pellitorine, and a furanoquinolines, γ-fagarine, from *Z. zanthoxyloides* roots, root-bark and stem-bark also inhibited 3D7 strains of *P. falciparum* with IC_50_ values of 2.2 and 2.0 μg/ ml, respectively (Goodman et al., 2019). It is possible that the anti-plasmodial compounds may have acted alone or together if present in the plant extracts used in treating malaria (Enechi et al., 2019; Amah et al., 2021). Despite the promising results, the mechanism of action of the isolated compounds are unknown and the research design in some cases did not include reference antimalarial agents for comparison. Future studies need to evaluate the clinical efficacy of the isolated anti-plasmodial compounds in susceptible populations as a treatment option for combatting resistant species of the malarial parasites.

## Potential Application of *Zanthoxylum* Species in Sickle Cell Disease

Sickle cell disease (SSD) is a group of genetic diseases resulting from inheritance of two abnormal copies of hemoglobin genes. The most common among them is sickle cell anemia. This disease is characterized by hemolytic anemia and occlusion of the blood vessels that reoccurs often. This occlusion is the cause of the excruciating crisis in the joints, a common occurrence in people with SSD. Upon hemolysis, hemoglobin in the erythrocytes is released as free heme (which is pro-inflammatory) and free iron (which by Fenton-type reaction interacts with hydrogen peroxide to form reactive oxygen species). These two components collectively worsen the complications associated with SSD. Consequently, there has been increased and continuous awareness on the prevention of SSD, and improvement in treatment regimen and other intervention strategies. People who cannot afford anti-sickling drugs like hydroxyurea, nitric oxide, purified poloxamer 118 and piracetam resort to medicinal plants with history of use in subsiding crisis associated with SSD (Okpuzor et al., 2008; Amujoyegbe et al., 2016). Medicinal plants have been investigated for anti-sickling activities and several have shown promising results. Notably, some members of genus *Zanthoxylum* (e.g., *Z. zanthoxyloides*, *Z. leprieuri* and *Z. gilletii*) are among the plants with history of traditional use in managing SSD (Akakpo-Akue et al., 2020). Some studies have reported the anti-sickling activities of *Z.* species and some anti-sickling compounds in these plant species have been isolated and characterized.

Among the species, *Z. zanthoxyloides*, *Z. lemairei, Z. leprieurii, Z. tessmannii* and *Z. gilletii* have been investigated for anti-sickling activity *in vitro* (Egunyomi et al., 2009; Ouattara et al., 2009). Moreover, the specific compounds responsible for anti-sickling activity of the plant extracts were scarcely reported. Particularly, three divallinoylquinic acids (burkinabins A, B, and C) isolated from *Z. zanthoxyloides* root bark at 1.964 mg/ ml inhibited sickling of deoxygenated erythrocytes by 77, 78.6 and 82.5% for burkinabins A, B, and C, respectively, which were similar to the effect of sodium chromoglycate, a reference anti-sickling agent (Ouattara et al., 2009). In addition, phenolic acids, such as syringic acid, vanillic acid, proto-catechuic acid, and *p*-hydroxy-benzoic acid, have been documented to play major roles in the anti-sickling activities of *Z. zanthoxyloides* (Nurain et al., 2017). However, this conclusion was only based on their identification in high amount in the active plant root extracts. Hence, bioassay-guided fractionation studies are needed to confirm the bioactivity of the phenolic acids, and to isolate other active compounds in the *Z.* species that showed anti-sickling activities. Generally, the ability of burkinabins A-C to demonstrate good anti-sickling properties has positioned the *Z.* species as promising sources of therapeutic agents for managing SSD. Clinical trials with the isolated anti-sickling compounds from *Z.* species are recommended and further studies are needed to modify the compounds to more potent and safer derivatives.

## Safety of *Zanthoxylum* Species

Knowing that not all things natural is safe, there is a need to be cautious in the use of natural products for food and drug. Many natural products have exhibited different levels of toxicity, including lethality at high doses (Al-Nuaimi 2018). For instance, *Z. chalybeum* root bark extract at 4,000 mg/ kg elevated serum creatinine, sodium and potassium levels in rats, as well induced histomorphological deterioration of the intestine in a manner consistent with tumor formation (Engeu et al., 2008). In addition, *in vivo* and *in vitro* studies showed that *Z. chalybeum* leaves, stem bark and root bark (2000 mg/ kg) caused mortality of mice and elicited toxicity against normal human renal epithelium cells. However, after solvent fractionation, no sign of toxicity was recorded at a maximum dose of 5,000 mg/ kg, suggesting that the toxic compounds might be acting in synergy. Furthermore, *Z. gilletii* stem bark applied in treating erectile dysfunction in South Africa and Peru was also reported to elicit histological changes in the reproductive system of male rat after oral administration daily for 14 days. Similarly, at high concentrations, *Z. zanthoxyloides* stem bark extract was genotoxic and cytotoxic against human leukocytes (Ogunbolude et al., 2014), while *Z. lepreurii* and *Z. zanthoxyloides* roots extracts were cytotoxic against normal human prostate epithelium cells (Tamdem 2019). Furthermore, *Z. zanthoxyloides* root bark induced seizure and substantial damage to the liver and kidney, resulting in mortality in mice that received large doses of the herbal materials; LD_50_ was recorded to be 5 g/ kg (Ogwal-Okeng et al., 2003). *Z. zanthoxyloides* stem bark was also reported to decrease bile release, affecting negatively the serum lipid levels of rats fed the extract (Umaru et al., 2019). In Ugandan folkloric practice, overdose of *Z. zanthoxyloides* has been recorded to cause short-duration and self-healing stomach disturbances (Anokbonggo et al., 1990). Lastly, *Z. heitzii* stem bark at doses higher than 6 g/ kg elicited toxicity and organ damage in rats (Ntchapda et al., 2015). Collectively, toxicities of the *Z.* species vary based on species, plant part, extraction solvent, dosage, level of phytochemical fractionation, and animal model studied. It is worth noting that the doses of the plant extracts that caused toxicity far exceed those that led to the desirable bioactivity. Nonetheless, caution should be taken to avoid consumption of high doses of the plant preparations to avoid deleterious effects. Furthermore, safe doses of some *Z.* species are yet to be reported and this information is necessary for traditional medicine practitioners to properly administer the natural products and for the safety of consumers.

## Conservation of *Zanthoxylum* Species for Future Applications

Considering the wide use of plants in the genus *Zanthoxylum* and the risk of extinction, conservationists have been advocating for measures to minimize overexploitation, especially for the species whose roots are the most commonly used part (Mbinile et al., 2020). For example, Ouédraogo et al. (2019) showed that the chemical constituents of the stem bark and root bark of *Z. zanthoxyloides* are similar; hence, extracts from these parts are likely to have similar biological activities. This observation needs to be confirmed by comparative phytochemomics and assessment of biological activities. If the relatedness is established, scientists and traditional practitioners would have sustainable alternatives and thus minimize overharvesting of the plant root. In addition, reforestation of medicinal plants and maintenance of their cell cultures for reuse should be emphasized (Li et al., 2020). Furthermore, genetic modification of medicinal plants to become more resilient to environmental threats, such as drought, will also help to make the species more sustainable.

It is worth noting that the names of some of the plants used in the studies reviewed were the “synonyms” as shown in plant databases. For example, some studies like Wu et al. (2007) and Fan et al. (2019) reported on *Zanthoxylum simulans* while Yang and Chen (2008), Yang et al. (2009), Chakthong et al. (2019), Sepsamli and Prihastanti, (2019) *,* Lu et al. (2020a) and Lu et al. (2020b) reported on *Zanthoxylum nitidum* instead of the accepted nomenclature, “Zanthoxylum bungeanum Maxim”. This issue was noted in several other papers, such as Chrian et al. (2011), Rodriguez-Guzman et al. (2011), Okafor et al. (2017), and Wansi et al. (2009). To address this issue, a recent study applied DNA barcoding for correct identification of plant species that were incorrectly named in previous studies (Veldmana et al., 2020). The authors noted that some medicinal plant researchers do not consult plant taxonomy experts and others do not confirm the plant identity by comparing their features with those available in reputable plant databases. Another possible source of this confusion is the local assignment of arbitrary names to many plant species by herbalists. This could also be associated with intraspecies and interspecies genetic diversity, and variance development because of changes in the environment (Feng et al., 2020). The issue could also be linked to the dependence of researchers in some countries on traditional medicine practitioners to provide and identify therapeutic plants. This system is not reliable. In one instance, some *Zanthoxylum* species with different chemical constituents and biological activities (*Z*. *bungeanum*, *Z*. *schinifolium*, and *Z*. *piperitum*) were distributed and intermixed as “*Zanthoxyli pericarpium*” by traditional medicine practitioners in Korea (Jang et al., 2020). Without proper identification, the outcome of research conducted with incorrectly labelled plant samples will be misleading. It is recommended that experts in phytotaxonomy should validate the identity of the plant species prior to further research and development. In addition, molecular characterization of the plant and the use of specific biomarkers may be helpful in ensuring that names given to plants used for phytopharmacological research are credible.

## Conclusion

**FIGURE 3:**
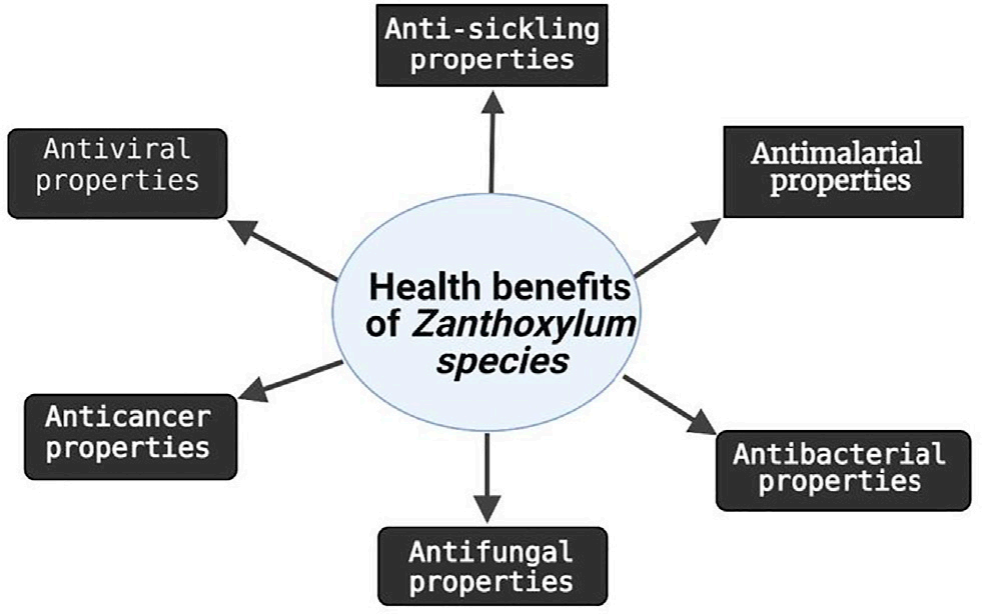
Summary of biological activities of extracts or compounds from different parts of *Zanthoxylum* species.

*Zanthoxylum* species are reservoir of phytochemicals with health-promoting properties, such as anti-sickling, anticancer and anti-infectious disease activities (Figure 3). The majority of the biological properties reported for *Z.* species were inspired by their traditional uses as therapeutic agents. Considering that the roots of *Z.* species are the most sourced parts in trado-medicinal uses, reforestation of the plants is highly recommended to avoid overharvesting. Similarly, the plant culture can be utilized instead of the freshly harvested plants. In many studies, drug-sensitive strains of infectious agents and cancer cells were used to assess bioactivity. Hence, future research should target the activity of *Z.* species against drug-resistant species or strains. This is important because one of the ultimate goals of new drug development is to curb drug resistance. Furthermore, several *Z.* species phytochemicals with strong bioactivities against infectious microorganisms, especially against drug-resistant strains of malarial parasites, viruses and other microbes, as well as drug-resistant cancer cells should be subjected to clinical trials as potential natural alternatives to synthetic drugs. In addition, since many studies were conducted *in vitro*, there is a dearth of information on the intestinal transport, biostability, bioaccessibility, and bioavailability of many of the active compounds. Additional research is needed to clarify the specific chemical compounds responsible for the promising biological activities of some of the plant extracts as well as the bioactivity and mechanisms of action of some of the isolated compounds. Future research should also confirm bioactivities and safety of the compounds *in vivo* using animal models and humans. Finally, researchers should endeavor to mimic the traditional methods used in preparation of the plant extract to ensure the preservation of the bioactive principles of interest.
